# Three levels of discrepancies in the records of trial sites in India, registered with the European Union Clinical Trials Register

**DOI:** 10.3389/fmed.2024.1357930

**Published:** 2024-07-05

**Authors:** Anwesha Dhal Samanta, Rishima Borah, Gayatri Saberwal

**Affiliations:** Institute of Bioinformatics and Applied Biotechnology, Bengaluru, India

**Keywords:** clinical trial registries, EUCTR, country of recruitment, data integrity, data quality, search strategies, trial registry metaresearch

## Abstract

**Introduction:**

Clinical trial registries serve a key role in tracking the trial enterprise. We are interested in the record of trials sites in India. In this study, we focused on the European Union Clinical Trial Registry (EUCTR). This registry is complex because a given study may have records from multiple countries in the EU, and therefore a given study ID may be represented by multiple records. We wished to determine what steps are required to identify the studies that list sites in India that are registered with EUCTR.

**Methods:**

We used two methodologies. Methodology A involved downloading the EUCTR database and querying it. Methodology B used the search function on the registry website.

**Results:**

Discrepant information, on whether or not a given study listed a site in India, was identified at three levels: (i) the methodology of examining the database; (ii) the multiple records of a given study ID; and (iii) the multiple fields within a given record. In each of these situations, there was no basis to resolve the discrepancy, one way or another.

**Discussion:**

This work contributes to methodologies for more accurate searches of trial registries. It also adds to the efforts of those seeking transparency in trial data.

## Introduction

Clinical trial registries are databases that serve a key role in keeping track of the trial enterprise, thereby promoting transparency and accountability in medical research. These registries were originally set up to meet two requirements. First, to list ongoing studies that patients may wish to participate in. And second, to avoid a bias in the literature that will arise if trial results are reported only if they are positive (1). If all trials are registered, it becomes difficult to hide those that do not yield a positive result. Aside from these two original aims, data in these registries have been used for many other purposes, such as tracking the advancement of cutting-edge science through trials, analyzing whether studies have been in compliance with the law, holding journals to account, analyzing the participation of developing country professionals in international studies, etc. (2).

Around the world, there are several public registries, and the prominent ones are ClinicalTrials.gov of the United States (US) and 17 registries that the World Health Organization (WHO) recognizes as Primary Registries (3). A given registry holds records for studies run in a country (such as Clinical Research Information Service, CriS, for the Republic of Korea), a region (the Pan African Clinical Trial Registry, PACTR, for all African nations), or in any part of the world (ClinicalTrials.gov). Clinical Trials Registry-India (CTRI) is one of the 17 primary registries. Mainly, CTRI holds records of studies that had sites in India, although it does accept records from countries that do not have their own Primary Registry (4).

Various stakeholders have pushed for the mandatory registration of all trials. Illustratively, since 2005, the International Committee of Medical Journal Editors, or ICMJE (5), has required the registration of each trial before the recruitment of the first participant. Also, there are laws in various countries or regions of the world that require that trials be registered. Examples include (a) the US’s Food and Drug Administration’s Amendments Act, or FDAAA, which, since 2007, has required the registration of a wide variety of studies (6), and (b) since 15 June 2009, it has been mandatory to register trials running in India with CTRI (7).

Our group is based in India and we are interested in the record of studies run locally. For instance, several of our studies have been concerned with how CTRI records could be improved (8–13).

Particularly relevant to this study is one where, using a modeling approach, we demonstrated that a few tens or hundreds of trials were not registered with CTRI, although the law required it, but were registered with ClinicalTrials.gov (9). This speaks to the issue of the “findability” of trials with sites in India.

In order to determine what steps need to be taken in order to identify every trial with sites in the country, we wished to study all major public registries. In unpublished work, we studied ClinicalTrials.gov. This is not a Primary Registry, but is the largest among these public registries, and is termed a data provider to WHO (14). Next, we wished to identify such trials that were registered in non-Indian Primary Registries. In unpublished work, we covered 15 of these registries but excluded the European Union Clinical Trial Registry (EUCTR). EUCTR is complex because a given study may have records from multiple countries in the EU, and therefore a given study ID may be represented by multiple records. This is illustrated by trial 2014-002275-28, which has records from 22 countries (15). Given this complexity, in this work we focused on EUCTR alone and determined what steps are required to identify the studies that listed sites in India that are in this registry.

## Methods

We provide an outline of the methods here, with further details in Supplementary material S1 and files referenced therein (Supplementary material S2–S7). Broadly, we used two methodologies. Methodology A involved downloading the EUCTR database and querying it for trials that had the keyword “India.” Since studies registered with CTRI have a trial ID beginning with “CTRI,” and such trials may be cross-referenced in EUCTR, we also queried EUCTR for studies containing “CTRI.” Methodology B used the search function on the website of EUCTR (16), searching for “India” and “CTRI.”

Each step in the Methodology was performed by two authors, independently.

## Results

**Figure 1:**
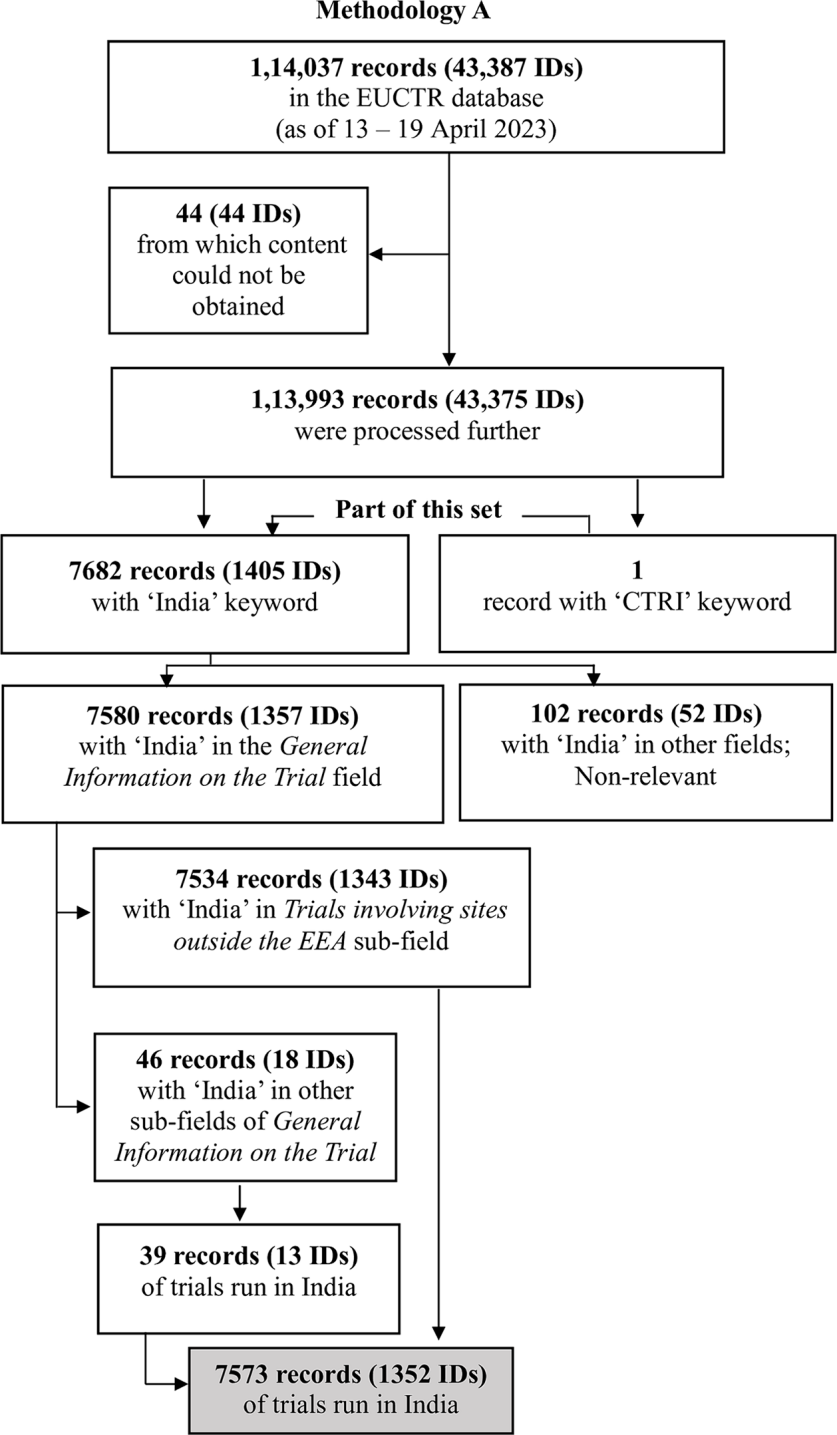
The steps used to identify the trials of interest by Methodology A.

**Figure 2:**
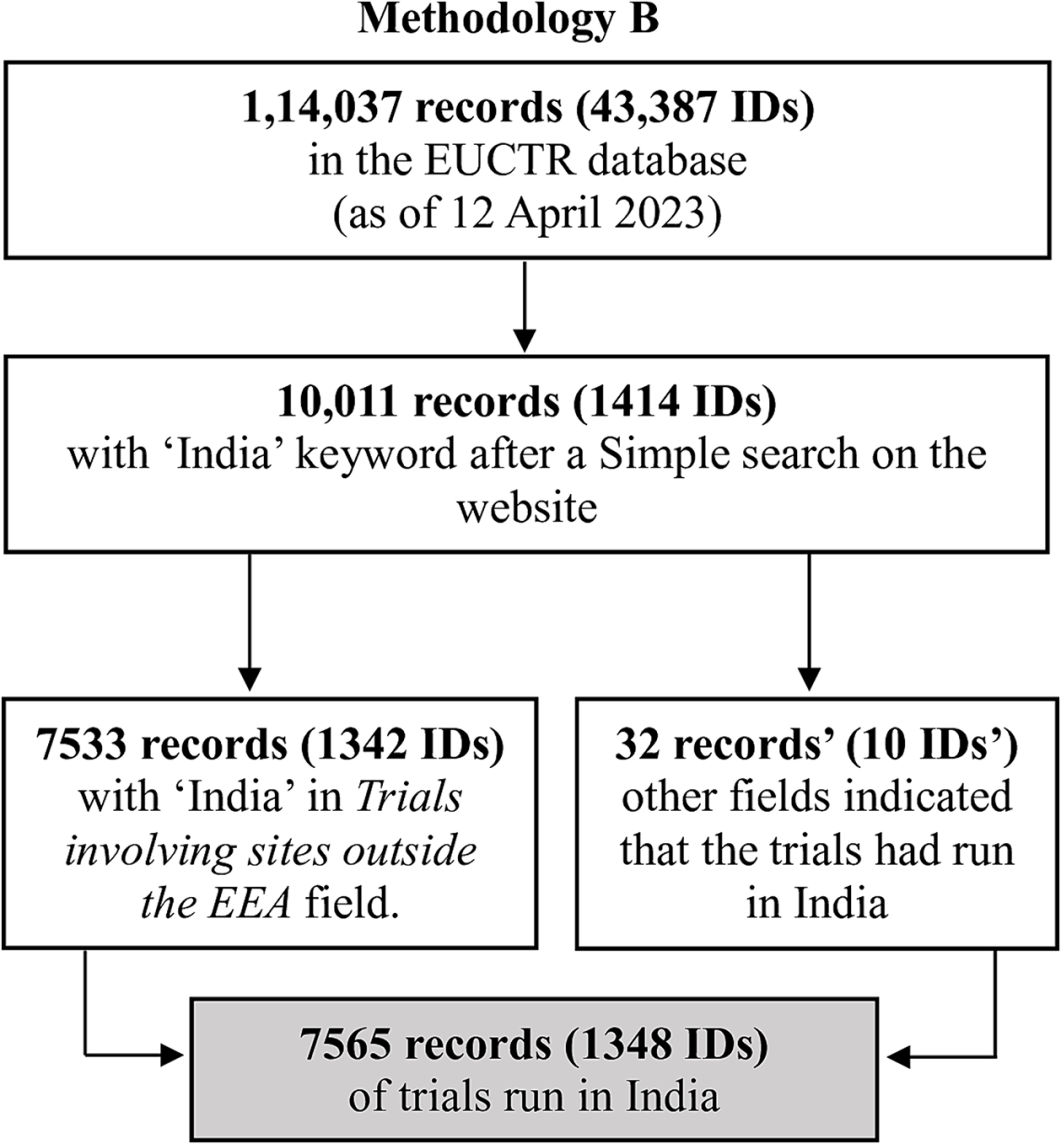
The steps used to identify the trials of interest by Methodology B.

The several steps used to identify the trials of interest by Methodologies A and B are outlined in Figures 1, 2, and are detailed in Supplementary material S1–S7.

Here, we summarize the key results obtained by the two methodologies:

Methodology A: This method yielded 7,534 records (with 1,343 unique IDs), all of which mentioned India in *Trial involving sites outside the EEA* (that is, outside the European Economic Area). There were 39 other records (13 IDs) that had information in other fields indicating that India had hosted the study. Overall, 7,573 records (1,352 IDs) indicated that the study had sites in India.
Methodology B: This method yielded 7,533 records (1,342 IDs) that listed India in *Trial involving sites outside the EEA*. There were 32 other records (10 IDs) that had information in other fields indicating that the trial had run in India. Overall, 7,565 records (1,348 IDs) indicated that the study had sites in India.

In summary, Methodology A identified four IDs (eight records) that Methodology B did not pick up.

These IDs were 2020-001335-28, 2008-007784-16, 2008-007762-39, and 2006-000156-40. In one case, 2020-001335-28-NL, India had been listed as a country of recruitment at the time of this work, although it is no longer listed. The other seven cases were as follows: 2008-007784-16, 2008-007762-39, and 2006-000156-40 (in the last in Sweden, Great Britain, Hungary, Germany, and Czechoslovakia). In each of these cases, no country was listed as a site of recruitment. However, India was listed elsewhere, in a manner that indicated that the trial had run in the country.

As a final step, for the 1,352 IDs identified by Methodology A, which included all those identified by Methodology B, we assessed how often India was mentioned in the multiple records per ID. Only 849 IDs (63%) had complete consensus on this matter, where all the records mentioned India. Other IDs had discrepant records. For example, trial ID 2021-005184-42 had four records, only one of which listed India (Supplementary material S8). As such, it had a discrepancy of three. The number of discrepancies ranged from 1 to 19 countries per ID, with the distribution captured in Table 1. The discrepancies have been decreasing over the years, from 2004 to 2022, and in a linear regression of years against the number of discrepant records, the best-fit line yields an *R*^2^ value of 0.7 (Supplementary material S8).

**Table 1:** For 1,352 EUCTR trial IDs, discrepancies between “The total number of countries per ID” and “The number of countries that listed India.”

S. No.	Discrepancies between “The total number of countries per ID” and “The number of countries that listed India”	Number of IDs	%
1.	0	849	62.8
2.	1–4	301	22.3
3.	5–9	160	11.8
4.	10–14	34	2.5
5.	15–19	8	0.6
		1,352	100

## Discussion

Our study was based on the idea that various stakeholders should be able to determine which clinical trials have run in the country. In parallel, this issue has come up in the United Kingdom (UK) as well. Following an important review of commercial clinical trials in the UK, the Lord O’Shaughnessy review recommended that the government of the UK should establish a single platform called clinicaltrials.gov.uk (17). This platform should track all phase 1 to phase 4 clinical trials conducted within the country. Our effort has been a more limited one, directed at identifying all trials running in India that have been registered with EUCTR. This follows our earlier work in which we posed the same question to the 15 other non-Indian Primary Registries.

We used two methodologies to search the EUCTR database for studies that had sites in India, and identified slight discrepancies in the numbers found by these two methods. As such, the most obvious way of looking for records of interest, Methodology B, may not provide the correct answer. Further, the discrepancies in the multiple records per study ID caused some confusion since there was no basis to decide which record was correct, that is, whether or not the trial had sites in India. Finally, in several records we found that another field, that one would not normally inspect in one’s search for relevant trials, indicated that the study had sites in India. The fact that the most relevant field, *Trial involving sites outside the EEA*, did not list India but that some other field did, is confusing. In summary, discrepant information was identified at three levels: (i) the methodology of examining the database; (ii) the multiple records of a given study ID; and (iii) the multiple fields within a given record. In each of these situations, there was no basis to resolve the discrepancy.

It is important that such discrepancies be resolved. Recently, it was pointed out that about half of biomedical research is not published (18), and therefore, at least for clinical trials, the data in registries becomes important. Nevertheless, speaking more broadly, there are, again, various levels at which one can fail to identify relevant data.

First, not finding the record at all. Several individuals and groups (19, 20) have been concerned with how to find all relevant trials. For instance, the Cochrane Collective recommends that even though ClinicalTrials.gov supplies data to the International Clinical Trial Registry Platform (ICTRP), one needs to search both ClinicalTrials.gov and ICTRP to find relevant records (21). However, that, too, may be inadequate (22).

Second, other researchers have found discrepancies in the records of a given study registered with more than one registry. Illustratively, in a 2018 study that looked at almost 10,500 studies that were registered with both ClinicalTrials.gov and EUCTR, one-third of the EU trials were found to have a “completion status” different from that in ClinicalTrials.gov (23). And in a 2021 study of almost 200 trials that were registered with multiple registries, primarily ClinicalTrials.gov and EUCTR, researchers found discrepancies in sponsors, funding sources, primary outcomes, sample size, etc. (24).

And third, aside from discrepancies across the records of a given study, previous work has described discrepancies within various fields of a given record (8). Illustratively, and most pertinently, in earlier work, we noted that discrepant data in the record of a trial registered with CTRI may cause confusion as to whether the study had run only in India or across multiple countries (including India) (8). In the current study, seven of the eight discrepant records concern India being listed in a field other than *Trial involving sites outside the EEA*. Nevertheless, Methodology B should have picked them up since it did pick up other cases where India was in fields other than the *Trial involving sites outside the EEA* field. We are not sure why this did not happen. It may have to do with the specific algorithm used by the search function. To be noted, these records pertained to the 2006–2008 time frame. Given that the number of discrepancies have come down over the years, the source of this particular discrepancy may have been addressed. We are also not sure why the record in 2020 was not picked up by the search function. Since India is no longer listed as a country of recruitment, it is possible that it was dropped in the small time difference between when we ran Methodology A and Methodology B.

Although tips for searching the EU trial database are available,^2^ this study provides a word of caution about using only the obvious Methodology B for interrogating a trial database and also for searching obvious fields for a given piece of information, in this case *Trial involving sites outside the EEA*. In order to accurately catalogues the studies that may have listed sites in India, we need to use the more cumbersome methodology of downloading the entire database and then check every study ID for which at least one record mentions India, and within such records, every field that mentions it.

Our findings echo those of other researchers who have found that using filters can exclude relevant studies, such as when interventional studies have been mislabeled as observational studies (18). As such, this work adds to efforts to provide methodologies for more accurate searches (21). It also adds to the efforts of those seeking transparency (25–26) in trial data. We note that information available in this database has been provided by the relevant national competent authorities, to whom the sponsor had provided the details. The European Medicines Agency, which manages the database, is not responsible for the veracity of the information inputted to its database (27). We also note that the database is not specifically designed to answer our research question. Nevertheless, it may be possible to provide further guidance to the sponsors or use logic rules that would decrease the occurrence of some of the discrepancies. Only accurate data meets the goals of transparency (28). And as a US Senator remarked, “Public-facing websites run by the government should be accurate. That’s not asking much.” (29).

### Limitations

In this work, we only studied the issue of whether or not a given study had sites in India. Although there may be discrepancies in other fields of data, we cannot extrapolate this work to those fields. Also, because we cannot verify information in a trial record with other documentation of the study, we cannot be sure of the veracity of all the inputted information.

Therefore, we may have both false positives (records indicating that India was a country of recruitment, although it was not) and false negatives (records that failed to indicate that India had been a country of recruitment, although it was). Finally, we have searched only for “India,” not for “IN” or for any misspellings of the word. It is possible that we missed records that did mention India, but in a different way.

In conclusion, in order to determine what steps need to be taken to identify every trial that listed sites in India that is registered with EUCTR, we used two methodologies to search the database. We also examined the multiple records, from multiple countries, of the same study ID. Finally, we examined not only the most obvious field, *Trial involving sites outside the EEA*, but also other fields for information that indicated that the study had sites in India. At all three levels there were discrepancies, which varied from small to large. It is unclear which data is reliable, and therefore it is important that the data be cleaned up and ways found to prevent such confusion in the future.

Transparency is meaningful only if the data is accurate.

## Data availability statement

The original contributions presented in the study are included in the article/Supplementary material, further inquiries can be directed to the corresponding author.

## Author contributions

ADS: Data curation, Formal analysis, Investigation, Methodology, Software, Validation, Visualization, Writing – review & editing. RB: Data curation, Formal analysis, Investigation, Methodology, Software, Validation, Visualization, Writing – review & editing. GS: Conceptualization, Formal analysis, Funding acquisition, Investigation, Methodology, Project administration, Resources, Supervision, Validation, Visualization, Writing – original draft, Writing – review & editing.

## Footnotes

^2^ https://euclinicaltrials.eu/search-tips-and-guidance/
