# Supplementary material for: Three levels of discrepancies in the records of trial sites in India, registered with the European Union Clinical Trials Register
Source: Front Med (Lausanne). 2024 Jul 5;11:1357930. doi: 10.3389/fmed.2024.1357930 (PMC11257842; doi:10.3389/fmed.2024.1357930)
Supplement: Supplementary file 1 [file Table_1.DOC]

**Supplementary File S1. Details of Methodologies A and B, and the results obtained from each.**

**Methodology A (based on downloading all records)**

**Step A: Downloading the records**

The records were downloaded from <https://www.clinicaltrialsregister.eu/ctr-search/search> between 13–19 April 2023 using script 1 (available in Box 1, at the end of this file). One ID may have had multiple associated records (each one representing a different European country), and therefore the number of IDs and the number of records was not the same. There were a total of 1,14,037 records with 43,387 associated unique IDs (detailed in Supplementary File S2.xls). This file includes the timestamp, that is the date and time for each downloaded page.

Of the 1,14,037 records, 44 (with 44 unique IDs) displayed the message “Problem Generating content! The trial requested was not found!” (detailed in Supplementary File S2.xls). Therefore, we processed the remaining 1,13,993 records with 43,375 unique IDs (Supplementary File S2.xls).

The break up of 43,387 IDs into 44 and 43,375 IDs, above, leads to a discrepancy of 32 IDs. This is explained below.

**Step B: Searching for the India keyword in the 1,13,993 records**

The 1,13,993 records had 43,375 IDs (Supplementary File S3.xls). From the set of the 44 records that displayed an error message, 32 IDs were a subset of these 43,375 IDs and the remaining 12 IDs were unique to the set of 44 IDs, thereby explaining the discrepancy mentioned above.

We web-scraped the 1,13,993 records using script 2 (available in Box 1, at the end of this file), with results available in Supplementary File S3.xls. The keyword ‘India’ (case-insensitive) was found in 7682 records, which corresponded to 1405 unique IDs. The remaining 1,06,311 records did not have ‘India’. Only 1 record had the keyword ‘CTRI’ and that record had both ‘CTRI’ and ‘India’ (case-insensitive for both). The ID of this record (2011-005586-21) was included in the 1405 IDs.

**Step C: Searching for the ‘India’ keyword in the field *General Information on the trial* in the 7682 records.**

In an EU record, the field *General Information on the trial* has sub-fields such as *Medical condition or disease under investigation, Objective of the trial*, *Design of the trial*, *Trial involving sites outside the EEA* and so on*.* The primary sub-field of interest was *Trial involving sites outside the EEA*. Therefore, we searched the 7682 records for the ‘India’ keyword in *General Information on the trial*. Among the 7682 records (1405 IDs), 7580 (1357 IDs) had ‘India’ in *General Information on the trial* (Supplementary File S4.xls)*.* The remaining 102 records (52 IDs) had ‘India’ in the fields *Protocol Information*, *Sponsors Information* and *IMP Identification* (Supplementary File S4 and Supplementary File S5.xls). Four IDs were common to the 1357 and 52 IDs.

In summary: 7682 records = 7580 + 102 records, and

1405 IDs = 1357 + 52 – 4 IDs

**Step D: Searching for ‘India’ in the sub-field *Trial involving sites outside the EEA* in the 7580 records:**

We web-scraped the 7580 records using script 3 (available in Box 1, at the end of this file). The results are available in Supplementary File S4.xls. Out of the 7580 records (1357 IDs), 7534 records (1343 IDs) had ‘India’ in the sub-field *Trials involving sites outside the EEA*. The remaining 46 records (18 IDs) had the keyword ‘India’ in other sub-fields such as *Principal inclusion criteria*, *Principal exclusion criteria* and so on. The details of these 46 records are available in Supplementary File S5.xls. There were four common IDs between the 1343 and 18 IDs (details in Supplementary File S4.xls).

In summary: 7580 records = 7534 + 46 records, and

1357 IDs = 1343 + 18 – 4 IDs

Even though ‘India’ was not mentioned in *Trial involving sites outside the EEA* in the above-mentioned 46 records, in the case of 39 records, descriptions in other fields indicated that the trial might have run in India (Supplementary File S5.xls).

In summary: 46 records yielded 39 relevant records, and

18 IDs yielded 13 relevant IDs

**Step E: Analyzing the 102 records that had ‘India’ keyword but were not present in *General Information on the trial*.**

These were the 102 records (52 IDs) in which ‘India’ was not present in *General information on the trial*. Among these 102 (details in Supplementary File S6):

- 69 records (37 unique IDs) had ‘India’ in *Sponsors Information*,
- 32 records (14 unique IDs) had ‘India’ in *IMP Identification*, and
- 1 record (1 unique ID) had ‘India’ in *Protocol* *Information*

In none of these 102 records did the descriptions indicate that the trial had run in India.

In summary: 102 records yielded 0 relevant records, and

52 IDs yielded 0 relevant IDs

**Results of Methodology A**

Number of records (and IDs) that indicated that the trial had run in India:

1. Those with ‘India’ in *Trial involving sites outside the EEA*: 7534 records (1343 IDs).
2. Those that were not listed in *Trial involving sites outside the EEA* but indicated that the trial had run in India: 39 records (13 IDs).

Therefore, number of trials that had run in India:

7534 + 39 records = 7573 records

1343 + 13 IDs = 1352 IDs (The number of IDs did not add up perfectly due to overlaps.)

**Methodology B (based on conducting a search on the registry website)**

This method involved the use of the search portal of the EUCTR website (<https://www.clinicaltrialsregister.eu/ctr-search/search>). We would have preferred to conduct an advanced search of the field ‘*Trial involving sites outside the EEA’*, but since there was no such option, we performed a simple search for the keyword ‘India’. This search was performed on 12 April 2023, and resulted in 10,011 records (1414 unique IDs). The links of these records were extracted using script 4 (available in Box 1, at the end of this file). These records were analyzed field-wise, as for Methodology A. The details are in Supplementary File S7.xls.

Out of the 10,011 records (1414 unique IDs), 7533 records (1342 IDs) had ‘India’ in the sub-field *Trials involving sites outside the EEA*. 32 records (10 IDs) had the keyword ‘India’ in other sub-fields such as *Principal inclusion criteria*, *Principal exclusion criteria* and so on.

In summary, the number of trials that had run in India:

7533 + 32 records = 7565 records

1342 + 10 IDs = 1348 IDs (The number of IDs did not add up perfectly due to overlaps.)

| **Box 1.** **Collated Scripts**  #********************* Script 1. Downloading all the records ************************  getwd()  setwd("D:/EU")  #to extract all the links from the website of eur registry and save them in a csv file.  library(rvest)  library(tidyverse)  eur <- data.frame()  for (i in seq(from=2134, to= 2142)) {  link <- url(paste0("https://www.clinicaltrialsregister.eu/ctr-search/search?query=&page=",i))  page <- read_html(link)  #ab <- page %>% html_nodes(".even+ tr:nth-child(3) td") %>% html_text()  ad <- page %>% html_nodes(".even+ tr a") %>% html_attr("href") %>% paste("https://www.clinicaltrialsregister.eu",., sep="")  eur = rbind(eur, data.frame(ad, stringsAsFactors = FALSE, check.rows = FALSE))  write.csv(eur,"eur1_links.csv")  print(paste("Page:", i))  }  #to use the csv file and download all the pages in html format  libraries = c("tidyverse", "stringr", "rvest", "xml2", "purrr", "data.table", "DBI", "httr")  lapply(libraries, require, character.only = TRUE)  #mydb <- dbConnect(RSQLite::SQLite(), "Download_Leb.sqlite") #connecting to a database that follows RSQLite  link <- data.frame()  link <- read.csv("eur1_links.csv")  link  i <- 1  ids <- c(1:6)  for (i in seq_along(ids)) {  official_url = link[ids[i],"ï..ad"]  print(official_url)  print(ids[i])    output_file=paste0("eu_page",ids[i],".html")  download.file(official_url, destfile = output_file, quiet = TRUE)      time_of_download = as.character(timestamp())  time_stamp = data.frame(Trial_ID = as.character(ids[i]),  downloaded_time = time_of_download,  URL = as.character(official_url))    write.table(time_stamp, "time_stamp_eu.csv", sep = ",",row.names = FALSE, col.names = !file.exists("time_stamp_eu.csv"), append = T)      counter = counter + 1  print(paste("Count = ", counter,"ID = ",ids[i]))    }  #***************** Script 2. Extracting the records with 'India' keyword ******************  libraries = c( "xml2","robotstxt", "tidyft","data.table", "DBI", "httr", "RSQLite","tidyverse","rvest","stringr","robotstxt","selectr","xml2","dplyr","forcats","magrittr","tidyr","ggplot2","lubridate","tibble","purrr","googleLanguageR","cld2")  lapply(libraries, require, character.only = TRUE)  ids <- c(1:114038)  counter = 0  for (i in seq_along(ids)) {    myfile = paste0("eu_page",ids[i],".html")    if (file.exists(myfile)) {    eu_page <- read_html(myfile)    if(is_empty(eu_page)) {    next  }    new_function <- function(a) {    if (length(a) == 0) {  a <- "NA"  } else if (a == "") {  a <- "NA"  } else {  return(a)  }  }    Trial_ID <- ids[i]  EduraCT_number <- eu_page %>% html_nodes("tr:nth-child(1) .cellLighterGrey") %>% html_text() %>% toString(.)  EduraCT_number <- new_function(EduraCT_number)  WP <- eu_page %>% html_nodes("#section-f .third , #section-e .third") %>% html_text() %>% str_squish() %>% str_trim()  WP_india <- toString(WP) %>% str_extract(., regex("\\bindia\\b", ignore_case = TRUE))  #WP_india_no_bound <- toString(WP) %>% str_extract(., regex("india", ignore_case = TRUE))  WP_ctri <- toString(WP) %>% str_extract(., regex("\\bctri\\b", ignore_case = TRUE))    India_ctri_EU <- data.frame(Serial_no,Reg_num,WP_india,WP_ctri)    write.table(India_ctri_EU,"India_ctri_eu.csv", sep = ",",row.names = FALSE, col.names = !file.exists("India_ctri_eu.csv"), append = T)    counter = counter + 1  print(paste("Count = ", counter,"ID = ",ids[i]))        }  }  #**********Script 3. Filtering the records with India in General Information field ***********  ids <- c(52:114037)  counter = 0  for (i in seq_along(ids)) {    myfile = paste0("eu_page",ids[i],".html")    if (file.exists(myfile)) {    eu_page <- read_html(myfile)    if(is_empty(eu_page)) {    next  }    new_function <- function(a) {    if (length(a) == 0) {  a <- "NA"  } else if (a == "") {  a <- "NA"  } else {  return(a)  }  }    Serial_no <- ids[i]    #WP <- eu_page %>% html_nodes("#section-f .third , #section-e .third") %>% html_text() %>% str_remove_all("\n") %>% str_remove_all("\r") %>% str_remove_all("\t") %>% str_trim() %>% str_squish()  #WP <- new_function(WP)    EudraCT <- eu_page %>% html_nodes("tr:nth-child(1) .cellLighterGrey") %>% html_text() %>% toString(.)  EudraCT <- new_function(EudraCT)    Date_of_Registration <- eu_page %>% html_nodes("tr:nth-child(6) .cellLighterGrey") %>% html_text() %>% str_remove_all("\n") %>% str_remove_all("\r") %>% str_remove_all("\t") %>% str_trim() %>% str_squish()  Date_of_Registration <- new_function(Date_of_Registration)    Trial_Status <- eu_page %>% html_nodes("tr:nth-child(5) .cellLighterGrey") %>% html_text() %>% str_remove_all("\n") %>% str_remove_all("\r") %>% str_remove_all("\t") %>% str_trim() %>% str_squish()  Trial_Status <- new_function(Trial_Status)    Trial_Results <- eu_page %>% html_nodes("tr:nth-child(7) .cellLighterGrey") %>% html_text() %>% str_remove_all("\n") %>% str_remove_all("\r") %>% str_remove_all("\t") %>% str_trim() %>% str_squish()  Trial_Results <- new_function(Trial_Results)    Member_state_concern <- eu_page %>% html_nodes("#section-a .tricell:nth-child(1) .third") %>% html_text() %>% str_remove_all("\n") %>% str_remove_all("\r") %>% str_remove_all("\t") %>% str_trim() %>% str_squish()  Member_state_concern <- new_function(Member_state_concern)    Clinical_Trial_Type <- eu_page %>% html_nodes("tr:nth-child(4) .cellLighterGrey") %>% html_text() %>% str_remove_all("\n") %>% str_remove_all("\r") %>% str_remove_all("\t") %>% str_trim() %>% str_squish()  Clinical_Trial_Type <- new_function(Clinical_Trial_Type)    Full_title_of_the_trial <- eu_page %>% html_nodes("#section-a .tricell:nth-child(3) .third") %>% html_text() %>% str_remove_all("\n") %>% str_remove_all("\r") %>% str_remove_all("\t") %>% str_trim() %>% str_squish()  Full_title_of_the_trial <- new_function(Full_title_of_the_trial)    WP <- eu_page %>% html_nodes("td") %>% html_text() %>% str_squish() %>% str_trim()  WP_india <- toString(WP) %>% str_extract(., regex("\\bindia\\b", ignore_case = TRUE))  WP_ctri <- toString(WP) %>% str_extract(., regex("\\bctri\\b", ignore_case = TRUE))    Summary_india <- eu_page %>% html_nodes(".cellLighterGrey") %>% html_text() %>% toString() %>% str_extract(.,regex("india",ignore_case = TRUE))  Protocol_india <- eu_page %>% html_nodes("#section-a .third") %>% html_text() %>% toString() %>% str_extract(.,regex("india",ignore_case = TRUE))  Sponsor_india <- eu_page %>% html_nodes("#section-b .third") %>% html_text() %>% toString() %>% str_extract(.,regex("india",ignore_case = TRUE))  IMP_india <- eu_page %>% html_nodes("#section-d .third") %>% html_text() %>% toString() %>% str_extract(.,regex("india",ignore_case = TRUE))  Information_on_placebo_india <- eu_page %>% html_nodes("#section-d8 .third") %>% html_text() %>% toString() %>% str_extract(.,regex("india",ignore_case = TRUE))  General_information_india <- eu_page %>% html_nodes("#section-e .third") %>% html_text() %>% toString() %>% str_extract(.,regex("india",ignore_case = TRUE))  Population_india <- eu_page %>% html_nodes("#section-f .third") %>% html_text() %>% toString() %>% str_extract(.,regex("india",ignore_case = TRUE))  Networks_india <- eu_page %>% html_nodes("#section-g .third") %>% html_text() %>% toString() %>% str_extract(.,regex("india",ignore_case = TRUE))  Ethics_india <- eu_page %>% html_nodes("#section-n .third") %>% html_text() %>% toString() %>% str_extract(.,regex("india",ignore_case = TRUE))  Third_country_india <- eu_page %>% html_nodes("#section-h .third") %>% html_text() %>% toString() %>% str_extract(.,regex("india",ignore_case = TRUE))  End_of_trial_india <- eu_page %>% html_nodes("#section-p .third") %>% html_text() %>% toString() %>% str_extract(.,regex("india",ignore_case = TRUE))    rr1 <- data.frame(Serial_no,EudraCT,Date_of_Registration,Trial_Status,Trial_Results,Member_state_concern,  Clinical_Trial_Type,Full_title_of_the_trial,WP_india,WP_ctri,Protocol_india,Sponsor_india,  IMP_india,Information_on_placebo_india,General_information_india,Population_india,  Networks_india,Ethics_india,Third_country_india,End_of_trial_india)    write.table(rr1,"India_all_info.csv", sep = ",",row.names = FALSE, col.names = !file.exists("India_all_info.csv"), append = T)    counter = counter + 1  print(paste("Count = ", counter,"ID = ",ids[i]))    }  }  #**** Script 4. Extracting all the links of the records that were generated by Methodology B ****  #to extract all the links from the website of eur registry having india as a keyword from basic search and save them in a csv file.  library(rvest)  library(tidyverse)  india_links_eu <- data.frame()  for (i in seq(from=1, to= 71)) {  link <- url(paste0("https://www.clinicaltrialsregister.eu/ctr-search/search?query=india&page=",i))  page <- read_html(link)  ab <- page %>% html_nodes(".even+ tr:nth-child(3) td") %>% html_text()  ad <- page %>% html_nodes(".even+ tr a") %>% html_attr("href") %>% paste("https://www.clinicaltrialsregister.eu",., sep="")    time_of_download = as.character(timestamp())  india_links_eu <- data.frame(ad,time_of_download)  write.table(india_links_eu, "time_stamp_eu_india_links.csv", sep = ",",row.names = FALSE, col.names = !file.exists("time_stamp_eu_india_links.csv"), append = T)    print(paste("Page:", i))  } |
| --- |
